# Ultrasonic Soldering of AlN/Cu Using SiC-Modified Zn5Al3Ti Active Solder

**DOI:** 10.3390/ma19132897

**Published:** 2026-07-06

**Authors:** Tomas Melus, Roman Kolenak, Mikulas Sloboda, Peter Gogola, Matej Pasak

**Affiliations:** Faculty of Materials Science and Technology in Trnava, Slovak University of Technology in Bratislava, Jana Bottu n. 2781/25, 917 24 Trnava, Slovakia; roman.kolenak@stuba.sk (R.K.); mikulas.sloboda@stuba.sk (M.S.); peter.gogola@stuba.sk (P.G.); matej.pasak@stuba.sk (M.P.)

**Keywords:** active solder, Zn-Al-Ti solder, SiC nanoparticles, AlN/Cu joint, ultrasonic soldering, microstructural characterization

## Abstract

This study investigates the effect of SiC nanoparticle addition on the microstructure, interfacial reactions, and mechanical properties of Zn5Al3Ti active solder used for ultrasonic soldering of AlN ceramic to copper substrates. Composite solders containing 3 and 6 wt.% SiC nanoparticles were prepared and applied under flux-free ultrasonic soldering conditions. The solder alloys were evaluated by tensile testing, while the soldered joints were evaluated by shear strength testing. The solder microstructure and interfacial regions were characterized using SEM/EDS analysis. The results showed that the addition of SiC nanoparticles modified the microstructure of the Zn5Al3Ti solder and influenced the mechanical performance of the ceramic/metal joints. Among the investigated systems, the AlN/Zn5Al3Ti + 6 wt.% SiC/Cu joint exhibited the highest shear strength, reaching approximately 101 MPa. SEM/EDS observations revealed the formation of compact multilayered interfacial regions, including possible Cu–Zn intermetallic phases at the Cu/solder interface and Al–Ti–Zn-based reaction products near the solder/AlN interface. The improved joint performance may be attributed to the combined effect of SiC-induced microstructural modification, the presence of Si-containing particles, and the formation of compact metallurgical bonds. The results indicate that Zn5Al3Ti solder modified with 6 wt.% SiC nanoparticles is a promising material for producing strong AlN/Cu joints under the applied ultrasonic soldering conditions.

## 1. Introduction

Ultrasonic soldering has proven to be an effective flux-free technique that utilizes acoustic cavitation and mechanical vibrations to remove oxide layers and enhance adhesion between the solder and the substrate. This technology has been successfully applied to joining various material combinations, such as Al/SiC, Al_2_O_3_/Cu, and Cf/Al composites [1,2,3,4]. Over the past decade, research on joining ceramic materials like Al_2_O_3_, AlN, ZrO_2_, and SiC to metallic substrates has increasingly focused on developing reliable joints. However, the poor wettability of ceramics and the mismatch in thermal expansion coefficients make it challenging to produce reliable joints with metals such as copper or aluminum [5]. Several approaches have been used for the fabrication of ceramic/metal joints, including active metal brazing, metallization followed by soldering or brazing, diffusion bonding, and direct-bonding technologies. In the case of AlN/Cu joints, these methods can provide reliable bonding; however, they often require high processing temperatures, additional metallization steps, vacuum or protective atmospheres, or carefully controlled interfacial reactions. Therefore, flux-free ultrasonic soldering using active solder represents a promising alternative, as ultrasonic activation can improve wetting and promote bonding at lower processing temperatures. For instance, studies on ultrasonic soldering of aluminium alloys have shown that increasing ultrasonic activation time can significantly improve mixing and reduce the fraction of brittle eutectic phases, thereby increasing shear strength. This was exemplified in the joining of 7034 aluminium alloy using Zn5Al and Zn5AlCu solder, where optimized activation times led to significant microstructural refinement and shear strengths reaching up to 249 ± 12 MPa. While the reduction in the brittle Zn-Al eutectic phase is beneficial for joint integrity, the research also cautions that excessive ultrasonic exposure (e.g., up to 60 s) can lead to a high-volume fraction of the α-Al phase, which may ultimately compromise mechanical performance due to increased brittleness [2]. Following the tightening of environmental regulations concerning the use of lead, the development of new lead-free solder has become a key direction in the electronic assembly industry [6,7,8,9,10].

While traditional tin-based types of solder are still widely used, their relatively low melting temperature limits their applicability in devices exposed to elevated temperatures, such as power modules [11,12]. Therefore, research attention has shifted toward zinc-based alloys, such as Zn-Al, Zn-Sn, and Au-Sn, which exhibit superior thermal stability [13,14,15,16,17,18]. Investigations into active solder, such as Zn5Al1.5Mg1.5Ti for Al_2_O_3_/Cu joining, confirmed that ultrasonic activation enhances wettability and promotes the formation of necessary interlayers at the interface [4]. Further research on Zn-In-Mg solder highlights that the microstructure and chemical composition of zinc-based solder play a crucial role in establishing a strong bond with the substrate. For instance, studies on joining metal–matrix composites to copper revealed that significant dissolution of the aluminium matrix occurs during joining, leading to a complex multiphase interfacial region containing intermetallic compounds such as Cu_3_._2_Zn_0_._7_Al_4_._2_ and Al(Cu,Zn)_2_. While these interfacial reactions are essential for bond formation, it was also observed that indium segregation along grain boundaries can substantially deteriorate the mechanical performance of the joint, emphasizing the need for precise control over the solder’s metallurgical evolution [19].

Recent studies have shown that the addition of nanoparticles to solder alloys represents an effective strategy for refining eutectic structures, suppressing the formation of brittle phases, and improving joint strength [1,4,5,20,21]. SiC nanoparticles appear particularly suitable due to their high hardness, thermal conductivity, and chemical stability. Their strengthening effect in metal matrices is commonly associated with dispersion strengthening, grain-refinement mechanisms, and the restriction of dislocation motion [21,22]. Their incorporation into Zn-Al-Ti solder may therefore contribute to improved microstructural stability and enhanced mechanical integrity of ceramic–metal joints.

Based on these findings, the present work focuses on investigating the effect of incorporating SiC nanoparticles into a Zn-Al-Ti active solder during ultrasonic soldering of AlN ceramic to a copper substrate. The aim of this work was to investigate the effect of SiC content on the microstructure, interfacial reaction layer structure, and mechanical properties of Zn5Al3Ti active solder used for the fabrication of AlN/Cu joints by ultrasonic soldering.

## 2. Materials and Methods

The solder alloy used in the experiment was prepared from the high-purity metallic elements Zn, Al and Ti, yielding active Zn5Al3Ti solder containing 5 wt.% Al and 3 wt.% Ti in a zinc-based matrix. In the present study, Zn5Al3Ti active solder containing 3 and 6 wt.% SiC nanoparticles was used in the soldering experiments. The selected SiC additions of 3 and 6 wt.% were based on our previous experience with the preparation of similar composite solder, for which these contents proved to be technologically feasible and sufficiently reproducible. At the same time, these levels allowed the effect of ceramic nanoparticles on solder microstructure and joint properties to be evaluated, while avoiding excessively low additions that would be difficult to dose accurately and disperse homogeneously, as well as excessively high additions that could negatively affect solder processability. The chemical composition of the solder was presented in Table 1.

The SiC nanoparticles were introduced into the Zn5Al3Ti solder during alloy preparation. A separate detailed characterization of the starting SiC powder was not performed in the present work; therefore, the exact particle size distribution and particle morphology are not discussed quantitatively. The dispersion state of the SiC-containing particles in the solder was evaluated indirectly by SEM/EDS analysis of the prepared solder microstructures. The SEM/EDS observations confirmed the presence of Si-containing particles distributed within the solder matrix; however, a completely uniform dispersion cannot be assumed. Local clustering or agglomeration of Si-containing particles may occur, particularly in the alloy containing 6 wt.% SiC. Therefore, the effect of SiC is discussed cautiously in terms of microstructural modification of the solder, rather than as a purely dispersion-strengthening effect.

Three types of ceramic substrates were used in the experiment and joined to copper (Cu) pads:

- Ceramic substrates (Al_2_O_3_, ZrO_2_, AlN) in the shape of a disc—Ø 15 × 3 mm,
- Cu (4 N, 99.99%) substrate in the shape of a disc—Ø 15 × 3 mm,
- Cu (4 N, 99.99%) substrate in the shape of a square with dimensions 10 × 10 × 3 mm.

**Figure 1:**
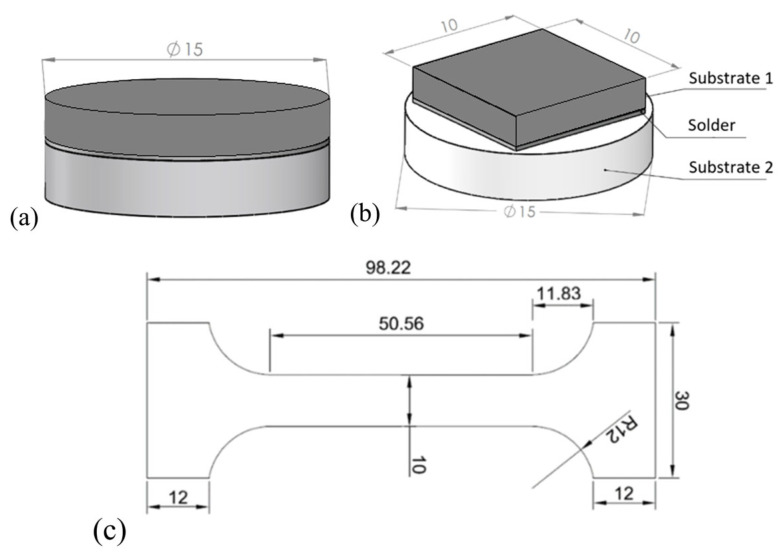
Configuration of the soldered joint: (**a**) for solder/substrate interface analysis; (**b**) for shear strength measurement [4]; (**c**) Test piece of a solder for static tensile strength test.

Tensile strength testing of the solder alloys was performed according to STN EN ISO 6892-1 [23] using a LabTest 5.250SP1-VM testing device equipped with a 250 kN load cell. The specimen dimensions were 4.2 × 10 × 1 mm, with an initial gauge length L0 of 80 mm. The test was carried out using a crosshead speed of 5 mm/min. The preload force was set to 50 N, and the test was terminated when the force decreased by 80% after reaching the maximum load. Three specimens were tested for each solder alloy composition. Soldering was carried out using an ultrasonic flux-free technique at a process temperature of approximately 420 °C. The joint configuration was designed for two types of evaluation: interfacial characterization of the solder/substrate interface, and measurement of the joint shear strength of metal-ceramic joints. A schematic representation of the sample arrangement was shown in Figure 1.

Ultrasonic activation was performed using a Hanuz UT2 ultrasonic converter with an oscillating piezoelectric system equipped with a titanium tool with a diameter of Ø3 mm. The key ultrasonic soldering parameters are listed in Table 2. The soldering temperature was monitored continuously using a NiCr/NiSi thermocouple. Ultrasonic energy was applied through the sonotrode during soldering, which facilitated disruption of the oxide layers on the ceramic substrates and activation of their surfaces for improved wettability.

**Figure 2:**
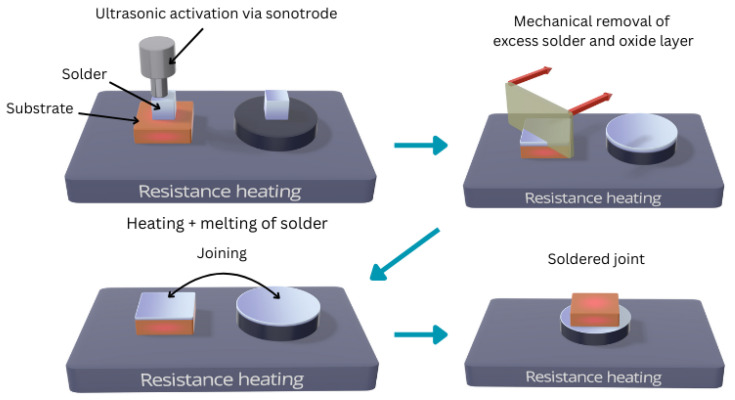
Schematic representation of the soldering procedure [4].

The ultrasonic parameters were kept constant for all experiments in order to compare the effect of SiC nanoparticle content and ceramic substrate type under identical soldering conditions. Soldering was carried out on a hot plate under simultaneous application of ultrasonic vibrations. Prior to soldering, both the AlN ceramic substrate and the Cu substrate were thoroughly degreased to remove surface contaminants. The substrates were then placed on a preheated hot plate, with the temperature set according to the melting range of the applied solder alloy (420 °C). A small amount of Zn5Al3Ti solder containing 3 or 6 wt.% SiC nanoparticles was applied onto the surface of the prepared substrate. After melting, the sonotrode of the ultrasonic device was immersed into the molten solder for 10 s. The ultrasonic action in the liquid solder enabled the removal of oxide layers and activation of the substrate surface, thereby improving its wettability. After ultrasonic activation, the copper substrate (Cu) was placed onto the ceramic substrate, forming the soldered assembly. Following the process, the sample was allowed to cool freely in air to room temperature. A schematic representation of the soldering procedure on a hot plate with the assistance of ultrasonic vibrations was shown in Figure 2.

Metallographic preparation of the cross-sections included sequential grinding and polishing of the surface. Grinding was performed on SiC abrasive papers with grit sizes of 240, 600, 1200 and 2400, with continuous water cooling throughout the process to remove generated grinding debris.

Final polishing of the samples was performed on rotary discs using diamond suspensions with particle sizes of 9 µm, 6 µm, 3 µm and 1 µm.

Microstructural evaluation was performed using a JEOL JSM 7600F scanning electron microscope (SEM/EDX, JEOL Ltd., Tokyo, Japan) equipped with a Schottky field emission electron source, operating at 20 kV and 90 µA. The samples were examined at a working distance of 15 mm using a backscattered electron detector. Elemental analysis was carried out using an Oxford Instruments X-Max silicon drift detector in combination with an energy-dispersive X-ray spectrometer (EDS, Oxford Instruments plc, Abingdon, UK).

## 3. Results

Two sets of samples were prepared for microstructure investigations and mechanical testing of all types of solder as well as the solder joints.

### 3.1. Evaluation of Tensile Strength of Solder Alloys

Tensile strength tests were performed to evaluate the effect of SiC nanoparticle addition on the mechanical behaviour of the Zn5Al3Ti solder alloy. Three solder alloy compositions were examined: the base Zn5Al3Ti solder, Zn5Al3Ti + 3 wt.% SiC, and Zn5Al3Ti + 6 wt.% SiC. For each composition, three specimens were initially tested in order to assess the repeatability of the results.

**Figure 3:**
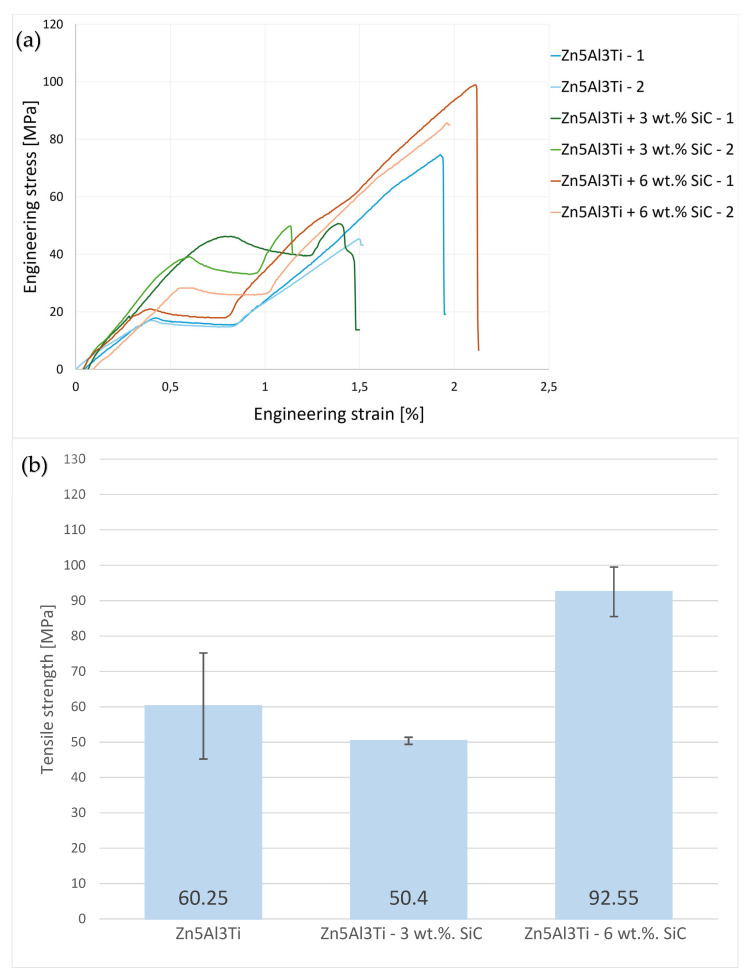
Mechanical behaviour of the investigated solder alloys after outlier screening: (**a**) engineering stress–strain curves used for the corrected evaluation; (**b**) corrected average tensile strength values with scatter.

Due to the scatter observed in the tensile strength data, the results were re-evaluated using an outlier-screening approach. The engineering stress–strain curves retained after screening are shown in Figure 3a, while the corrected average tensile strength values are shown in Figure 3b. The individual tensile test results retained after this screening are listed in Table 3.

Based on the screened results, the base Zn5Al3Ti solder reached a corrected average tensile strength of 60.25 MPa. The solder modified with 3 wt.% SiC reached a corrected average tensile strength of 50.4 MPa, indicating that this SiC addition did not improve the tensile strength. In contrast, the Zn5Al3Ti + 6 wt.% SiC solder exhibited the highest corrected average tensile strength, reaching 92.55 MPa. This improvement may be associated with the more pronounced microstructural modification caused by the higher SiC nanoparticle content.

In addition to the tensile strength values, the elongation values obtained from the tensile tests were also considered in order to better describe the mechanical behaviour of the investigated solder alloys. All examined types of solder exhibited low elongation values, generally below 1.2%, indicating limited plastic deformation before fracture. This behaviour is characteristic of the investigated Zn-based solder alloys and is associated with their relatively brittle response under tensile loading.

### 3.2. Shear Strength Evaluation of Soldered Joints

**Figure 4:**
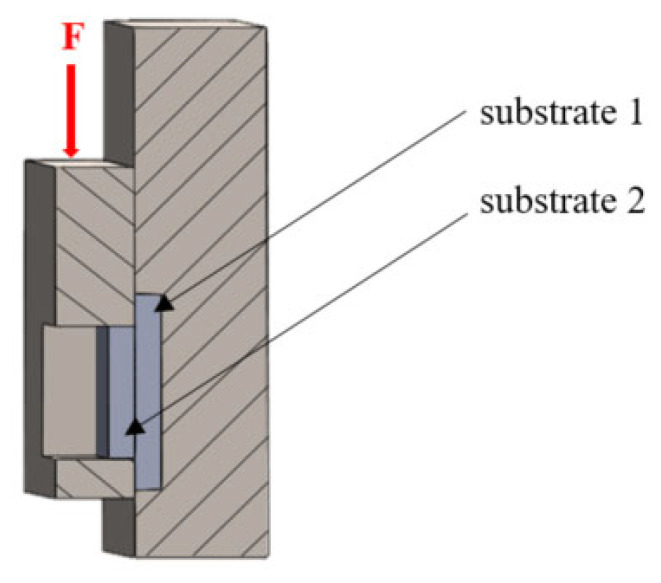
Schematic representation of the fixture for shear strength testing [4].

Soldered joints are subjected to thermomechanical loading during service due to thermal fluctuations and shear forces. The examined joints were tested for shear strength using a LabTest 5.250SP1-VM testing device, and the obtained values were compared. Shear strength was measured using a dedicated fixture that converted the tensile force into shear loading. The load was applied uniformly in the plane of the solder/substrate interface over a 10 × 10 mm bonded area, as schematically shown in Figure 4. The test was performed until complete failure of the specimens at a loading rate of 2 mm/min. Two specimens were tested for each joint combination.

**Figure 5:**
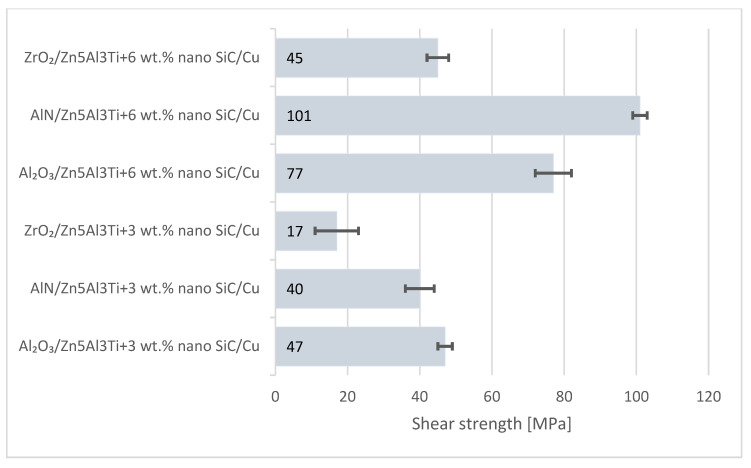
Average shear strength values of the experimental soldered joints with standard deviation.

The average shear strength values of the individual soldered joints are shown in Figure 5. The measurements were performed on joints produced using Zn5Al3Ti solder with 3 wt.% and 6 wt.% SiC nanoparticles on Al_2_O_3_, AlN, and ZrO_2_ ceramic substrates joined to copper. The results indicate that both the substrate type and the quantity of added nanoparticles affected the resulting shear strength of the joints.

The highest value is obtained for the AlN/Zn5Al3Ti + 6 wt.% SiC/Cu joint, where the average shear strength reaches approximately 101 MPa. In contrast, the lowest strength is recorded for the ZrO_2_/Zn5Al3Ti + 3 wt.% SiC/Cu joint, with a value of 17 MPa. A similar qualitative trend is also observed for the Al_2_O_3_ and ZrO_2_ substrates, where the joints prepared with 6 wt.% SiC generally exhibited higher shear strength than those prepared with 3 wt.% SiC, although the absolute values remained lower than those obtained for the AlN/Cu system.

The differences in shear strength between the investigated ceramic substrates can be explained by the combined influence of thermal expansion mismatch, wettability, and interfacial reaction behavior. During cooling from the soldering temperature, residual stresses may develop in the ceramic/solder/Cu assembly due to the different coefficients of thermal expansion of the ceramic substrates, solder alloy, and copper. These stresses can weaken the interface or promote crack initiation, especially when the interfacial reaction layer is not sufficiently compact and uniform.

However, the observed strength trend cannot be attributed only to thermal expansion mismatch. The highest shear strength obtained for the AlN/Zn5Al3Ti + 6 wt.% SiC/Cu joint suggests that AlN exhibited more favorable compatibility with the active Zn5Al3Ti solder under the applied ultrasonic soldering conditions. This can be associated with improved wettability and the formation of a compact interfacial region, as confirmed by SEM/EDS analysis. The lower strength values obtained for Al_2_O_3_/Cu and especially ZrO_2_/Cu joints may be related to less effective wetting and/or less continuous interfacial reaction layers. Therefore, the final mechanical performance of the joints was determined by the combined effect of SiC nanoparticle content, ceramic substrate type, wettability, interfacial bonding quality, and residual stresses generated during cooling.

Although the corrected tensile strength results and the shear strength results show a similar qualitative tendency, especially for the solder containing 6 wt.% SiC, these two mechanical responses cannot be directly correlated in a quantitative manner. The tensile test describes the behaviour of the bulk solder alloy, whereas the shear test reflects the response of the complete ceramic/solder/Cu joint. Therefore, the shear strength is affected not only by the mechanical properties of the solder, but also by the ceramic substrate type, wettability, interfacial reaction layer formation, interfacial bonding quality, and residual stresses generated during cooling. In addition, only two shear specimens were tested for each joint combination, which is not sufficient for reliable statistical outlier screening or for establishing a predictive tensile–shear relationship.

A Hall–Petch-type relationship was also not introduced because the eutectoid lamellar spacing was not quantitatively measured and statistically evaluated in the present study. Although refinement of the eutectoid lamellar mixture with increasing SiC content may contribute to the observed mechanical behaviour, the available microstructural data are qualitative and therefore do not provide a sufficient basis for formulating a reliable modified Hall–Petch equation.

### 3.3. Analysis of Zn–5Al–3Ti Alloy and Its Modifications with SiC Nanoparticles

**Figure 6:**
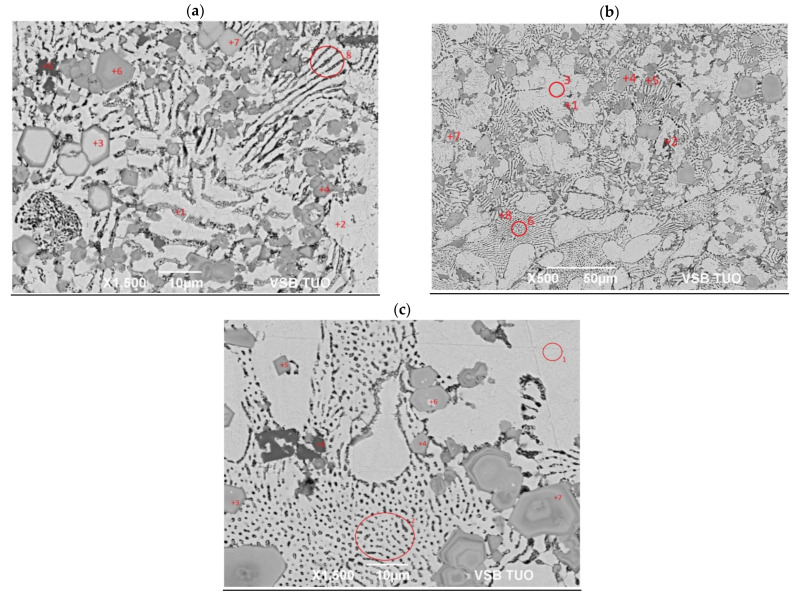
Point EDX analysis of the microstructures of the individual types of solder: (**a**) Zn5Al3Ti alloy (**b**) Zn5Al3Ti + 3 wt.% SiC nanoparticles (**c**) Zn5Al3Ti + 6 wt.% SiC nanoparticles.

Based on point EDX analysis, possible phases in the microstructures of all examined solders were assigned, with the analysed locations marked in the microstructural images (Figure 6a–c) and their chemical compositions listed in Table 4, Table 5 and Table 6. Three material variants were analysed: the base Zn5Al3Ti alloy, the alloy with 3 wt.% SiC nanoparticles, and the alloy with 6 wt.% SiC nanoparticles.

The microstructure of the Zn–Al–Ti solder is partly governed by phase relations in the binary Al–Zn system. In particular, the Zn-rich matrix corresponds to a Zn-based solid solution with limited Al solubility, while the lamellar regions are associated with the eutectoid transformation forming the (Al) + (Zn) mixture. Since the studied solder also contains Ti and, in the modified variants, SiC nanoparticles, the final phase constitution must be interpreted with respect to the multicomponent nature of the system. For clarity, the nominal solder compositions are given in wt.%, whereas the local EDS results used for phase identification are reported in at.%.

**Figure 7:**
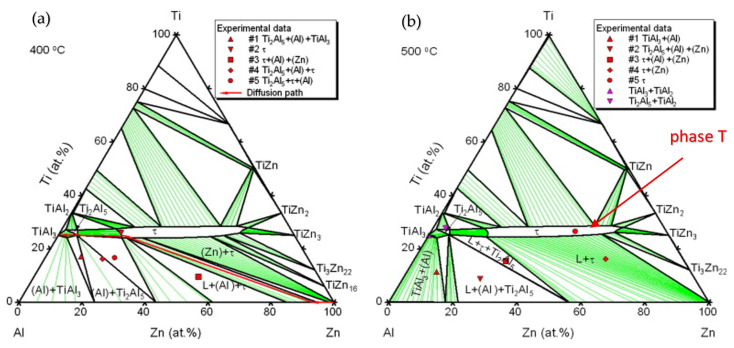
Isothermal ternary phase diagrams of the Al–Zn–Ti system: (**a**) at 400 °C; (**b**) at 500 °C [24].

The microstructure of the Zn5Al3Ti alloy (Figure 6a, Table 4) is characterized by phases typically formed in the Zn-Al-Ti system. In the eutectoid regions, the measured aluminium and zinc concentrations correspond to the (Al) + (Zn) eutectoid mixture that forms in the Al-Zn system at 277 °C. The lamellar structures consist of alternating brighter zinc-rich and darker aluminium-rich lamellae, confirming the eutectoid transformation. In the bright areas of the microstructure, nearly pure zinc with a minimal amount of aluminium was detected, representing the (Zn) solid solution. Polygonal particles dispersed within the matrix exhibited a Zn:Ti atomic ratio of approximately 70:30, which corresponds to the binary intermetallic phase Zn_3_Ti. In addition, irregular particles containing Al, Ti, and Zn were detected, with compositions located in the region of the ternary intermetallic T-phase (Figure 7). The phase morphology and distribution are consistent with reported data for the Zn-Al-Ti system [22].

The microstructure of the Zn5Al3Ti alloy with the addition of 3 wt.% SiC nanoparticles (Figure 6b, Table 5) shows distinct changes compared with the base alloy. This modified alloy exhibits the same primary phases as the unmodified solder, but with an increased presence of silicon. In the eutectoid regions, the lamellar (Al) + (Zn) two-phase structure was again identified. The analysed points contained combinations of Al, Ti, Zn and Si, where silicon was predominantly detected in dark particles representing the SiC carbides. Slight variations in concentration were observed locally, caused by partial overlap of the EDX beam with the surrounding Al-rich matrix. The bright areas represented the (Zn) solid solution with a low aluminium content. Grey particles contained Al, Ti and Zn in a ratio corresponding to the (Zn,Al)_3_Ti phase. Irregular dark areas may correspond to the ternary intermetallic T-phase. The composition in spectrum location 7 suggests the possible presence of Zn_3_Ti (≈26:74 at.% Ti:Zn). Other analysed points again showed a composition characteristic of the T-phase, this time with a slightly reduced zinc content.

The microstructure of the Zn5Al3Ti alloy with the addition of 6 wt.% SiC nanoparticles (Figure 6c, Table 6) exhibits a more pronounced modification effect compared to the alloy containing 3 wt.% SiC. With the increased SiC content, the microstructure retains the same phase types as the lower-modified solder, but with a higher concentration of Si- and Ti-rich particles. The bright regions consist of almost pure zinc with a small amount of dissolved aluminium. Adjacent lamellar structures of (Al) + (Zn) represent the typical eutectoid formed below 277 °C.

Polygonal grey particles contained Al, Zn, and Ti corresponding to the ternary T-phase, as well as the (Zn,Al)_3_Ti phase. The measured composition suggests the possible presence of the binary Zn_3_Ti phase, with measured values of 27.5 at.% Ti and 72.5 at.% Zn closely matching its stoichiometry. Fine, dark particles with high Si and Ti content may correspond to a Si–Ti-rich phase or Si–Ti-type region.

Overall, the microstructure of this alloy consists of a combination of primary (Zn) solid solution, eutectoid lamellar mixtures of (Al) + (Zn), ternary T-phase, binary Zn_3_Ti phase, and locally present Si–Ti-rich regions. The spatial distribution of these phases corresponds well to the chemistry of the Zn-Al-Ti solder modified with SiC nanoparticles [4,22].

### 3.4. Analysis of the Interfacial Zone Between Cu/Zn5Al3Ti + 6 wt.% SiC

**Figure 8:**
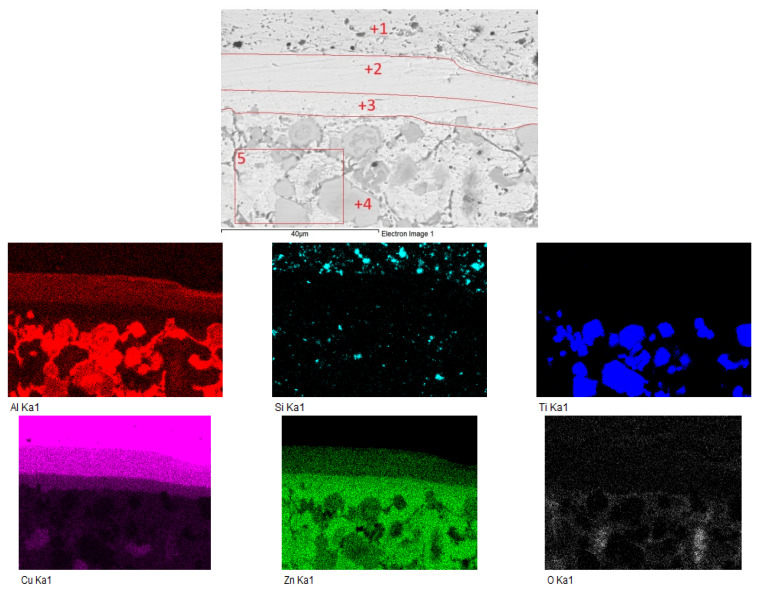
SEM backscattered electron image of the interfacial region between Cu and the Zn5Al3Ti + 6 wt.% SiC nanoparticle solder, showing the locations selected for EDS analysis. Spectra 1–4 correspond to point analyses marked in the SEM image, while Spectrum 5 corresponds to the boxed area analysis. Elemental maps show the distribution of O, Al, Si, Ti, Cu, and Zn.

The AlN/Zn5Al3Ti + 6 wt.% SiC/Cu joint exhibited the highest shear strength and was therefore selected for interfacial analysis. Figure 8 shows the elemental distribution across the interface between the copper substrate and the Zn5Al3Ti + 6 wt.% SiC solder, including the locations selected for point EDS analysis (Spectra 1–5). The elemental maps reveal a multilayered interfacial structure. The corresponding EDS results and tentative region or phase assignments are summarized in Table 7.

In the upper region (Spectrum 1), Cu was the dominant element, with 85.99 at.% Cu, while minor amounts of O (4.21 at.%), Al (0.56 at.%), Si (6.85 at.%), and Zn (2.40 at.%) were also detected. Ti was not detected in this region. This composition corresponds mainly to the Cu substrate, with minor contributions from neighbouring interfacial or solder regions.

Spectrum 2 contained 10.32 at.% Al, 36.36 at.% Cu, and 51.47 at.% Zn, with a small amount of O (1.86 at.%), while Si and Ti were not detected. This composition indicates the formation of a Cu–Zn intermetallic region, possibly corresponding to Cu_5_Zn_8_ or CuZn, with minor Al contribution.

In the subsequent region (Spectrum 3), 66.88 at.% Zn, 23.48 at.% Cu, 6.41 at.% Al, 0.44 at.% Si, and 2.80 at.% O were detected, while Ti was not detected. This composition indicates progressive Zn enrichment toward the solder and suggests the presence of a Zn-rich interfacial region, which may correspond to a mixture of ε(CuZn_4_) and Zn-rich solid solution.

Within the interfacial layer (Spectrum 4), 27.49 at.% Al, 22.34 at.% Ti, and 44.81 at.% Zn were measured, together with minor amounts of O (4.29 at.%), Si (0.69 at.%), and Cu (0.38 at.%). This composition suggests the formation of an Al–Ti–Zn interfacial reaction region. Based on the simultaneous presence of Al, Ti, and Zn, this region may locally correspond to an (Al,Ti)Zn_2_-type phase; however, this phase assignment should be considered tentative because it is based only on SEM/EDS analysis.

The area analysis of the solder region (Spectrum 5) measured 15.27 at.% Al, 1.84 at.% Si, 6.79 at.% Ti, 3.18 at.% Cu, and 59.66 at.% Zn, together with 13.27 at.% O. This composition corresponds to the Zn5Al3Ti solder region containing Si-containing particles. The detected Si in this region indicates the presence of Si-containing particles within the analysed solder area, while the detected oxygen may be related to surface oxidation or local oxide residues.

The formation of a compact Cu–Zn–Al–Ti interfacial region indicates good metallurgical bonding between the Cu substrate and the solder. This compact interfacial structure is consistent with the high shear strength measured for the AlN/Zn5Al3Ti + 6 wt.% SiC/Cu joint.

### 3.5. Analysis of the Interfacial Region Between Zn5Al3Ti + 6 wt.% SiC/AlN

**Figure 9:**
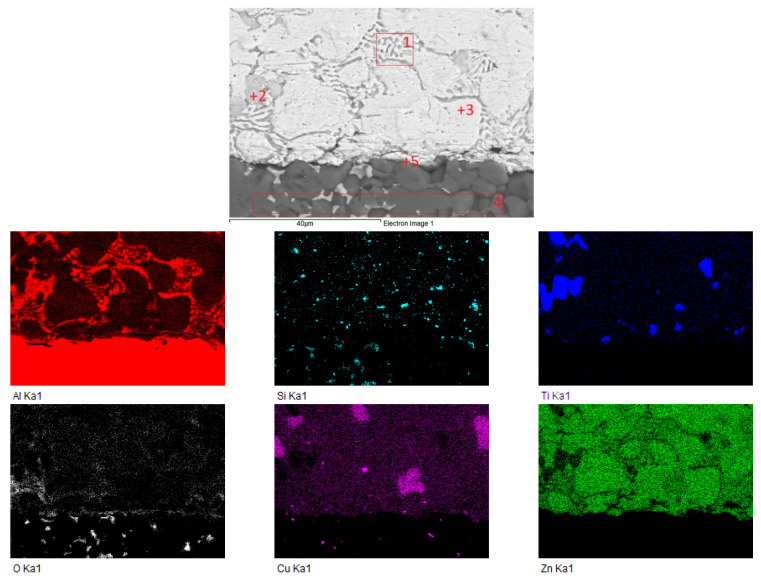
SEM backscattered electron image of the interfacial region between AlN and the Zn5Al3Ti + 6 wt.% SiC nanoparticle solder, overlaid with EDX analysis points 1–5. Elemental maps show the distribution of O, Al, Si, Ti, Cu, and Zn.

Figure 9 shows the planar elemental distribution in the interfacial region between the AlN ceramic substrate and the Zn5Al3Ti + 6 wt.% SiC solder, including the locations of point EDS analyses (Spectra 1–5). The elemental maps indicate that the interface exhibits a compact character without pores or cracks, confirming good wettability of the solder on the ceramic substrate and the formation of a strong metallurgical bond between AlN and the metal solder. The corresponding EDS results and tentative region or phase assignments are summarized in Table 8.

Spectrum 1 contained 6.20 at.% Al, 0.71 at.% Si, 0.76 at.% Ti, 4.07 at.% Cu, 84.44 at.% Zn, and 3.81 at.% O, while N was not detected. This composition corresponds to a Zn-rich region of the solder matrix. In contrast, Spectrum 2 contained 12.21 at.% Al, 0.27 at.% Si, 19.45 at.% Ti, 0.42 at.% Cu, 66.14 at.% Zn, and 1.50 at.% O, while N was not detected. This composition indicates the presence of an Al–Ti–Zn interfacial reaction region.

The region corresponding to Spectrum 3 contained 3.05 at.% Al, 0.36 at.% Si, 3.53 at.% Cu, 90.72 at.% Zn, and 2.34 at.% O, while Ti and N were not detected. This composition represents a Zn-based solder solid solution. In Spectrum 4, the point EDS analysis measured 65.17 at.% Al and 29.65 at.% N, with minor amounts of Cu (0.20 at.%), Zn (0.33 at.%), and O (4.65 at.%), while Si and Ti were not detected. These values are consistent with the AlN substrate.

In the interfacial layer near the AlN substrate (Spectrum 5), the point EDS analysis measured 35.79 at.% Al, 0.28 at.% Si, 3.75 at.% Cu, 52.34 at.% Zn, and 7.83 at.% O, while Ti and N were not detected. The high Al and Zn contents suggest the formation of an Al–Zn-based interfacial reaction region near the AlN substrate, with minor contributions of Si, Cu, and O.

Based on the simultaneous presence of Al, Ti, and Zn in Spectrum 2, the formation of an Al–Ti–Zn interfacial reaction region on the solder-side interface can be suggested. Possible intermetallic phases such as (Al,Ti)Zn_2_ or Al_3_Ti may occur locally; however, this assignment should be considered tentative because it is based only on SEM/EDS analysis.

Although Si was detected by EDS in the solder and near the interfacial region, no continuous Si-rich reaction layer was observed at the solder/AlN interface. The detected Si is therefore mainly attributed to Si-containing particles dispersed in the solder matrix. In regions where Si and Ti were detected simultaneously, the local formation of a Si–Ti-type phase may be considered. However, based on the present SEM/EDS results, there is no clear evidence that SiC nanoparticles directly participate in the formation of the main interfacial reaction layer between the solder and the AlN substrate. Their effect is therefore more likely related to the modification of the solder microstructure. Since EDS analysis alone cannot unambiguously distinguish retained SiC particles from possible Si-containing reaction products, this interpretation should be considered qualitative.

### 3.6. Fracture Surface Analysis of the AlN/Zn5Al3Ti + 6 wt.% SiC/Cu Joint

To clarify the failure mode of the joint with the highest shear strength, fracture surface analysis was performed on the AlN/Zn5Al3Ti + 6 wt.% SiC/Cu joint after shear testing. The SEM image of the fracture surface shows a rough, irregular and highly heterogeneous morphology with adhered particles and locally deformed regions. This morphology indicates non-uniform crack propagation during mechanical loading.

**Figure 10:**
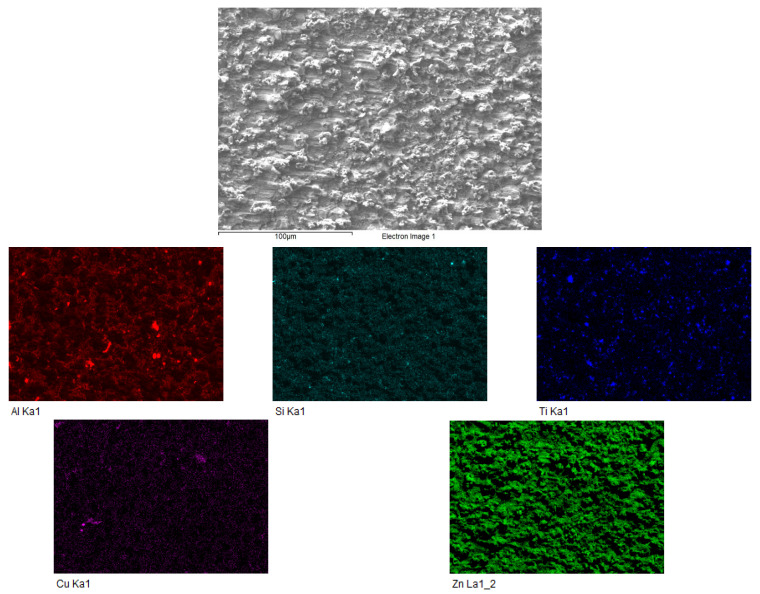
SEM image and EDS elemental maps of the fracture surface of the AlN/Zn5Al3Ti + 6 wt.% SiC/Cu joint after shear testing, showing the distribution of Zn, Ti, Cu, Si and Al.

EDS elemental mapping of the fracture (Figure 10) surface revealed that Zn and Al were the dominant elements distributed over the analysed area, while Cu, Ti and Si were detected mainly locally. The pronounced Zn distribution, together with the presence of Al-rich regions, indicates that a significant part of the fracture surface was covered by the solder alloy. This suggests that failure did not occur exclusively as a clean separation at the AlN/solder interface. Instead, crack propagation probably occurred mainly within the solder layer and/or near the interfacial region. The local presence of Ti and Si may be associated with intermetallic phases and Si-containing particles present in the modified solder.

These observations support the conclusion that the AlN/Zn5Al3Ti + 6 wt.% SiC/Cu joint formed a relatively strong interfacial bond, consistent with its high measured shear strength. Based on the SEM fractography, clearly developed ductile dimples were not observed at the investigated magnification. Therefore, the failure mechanism may be described cautiously as predominantly brittle or quasi-brittle, with limited local ductile deformation.

## 4. Conclusions

The aim of this work was to evaluate the effect of SiC nanoparticles on the mechanical properties of the ultrasonically soldered Zn5Al3Ti alloy and to assess its suitability for joining an AlN ceramic substrate to a Cu substrate. The study focused primarily on analysing the shear strength of the soldered joints and the microstructure of the solder at different concentrations of SiC nanoparticles (3 and 6 wt.%). The obtained results can be summarised as follows:

- Ultrasonic soldering enabled the formation of a joint between the AlN ceramic substrate and the copper substrate, promoting improved contact between the solder and the ceramic surface. Microstructural analysis showed that the addition of SiC nanoparticles modified the solder microstructure compared to the base Zn5Al3Ti alloy.
- The shear strength of the joints was affected by both substrate type and the amount of SiC nanoparticles. The highest value, approximately 101 MPa, was measured for the AlN/Zn5Al3Ti + 6 wt.% SiC/Cu system. Compared with the corresponding AlN/Cu joint prepared using the lower SiC content, a substantial increase in shear strength was observed.
- Microstructural (SEM/EDS) analysis suggested the possible formation of a compact multilayered interfacial region without pores or cracks. At the Cu/solder interface, possible Cu–Zn intermetallic phases, such as Cu_5_Zn_8_ and CuZn, may be present, while at the solder/AlN interface, Al_3_Ti and (Al,Ti)Zn_2_ phases may occur.
- SEM/EDS observations indicated the presence of Si-containing particles within the solder matrix. Their contribution to the mechanical performance of the joint may be associated with microstructural modification and interfacial phase development. The assessment of particle distribution in this work is based on qualitative observations from the analysed regions.
- The improved mechanical performance of the joint with 6 wt.% SiC may be associated with SiC-induced microstructural modification of the solder, the presence of Si-containing particles, and the formation of compact interfacial reaction layers. However, based on the present SEM/EDS results, the dominant strengthening mechanism cannot be unambiguously identified.
- Based on the obtained results, it can be concluded that the Zn5Al3Ti solder with the addition of 6 wt.% SiC is suitable for joining AlN ceramics to a Cu substrate, forming strong and compact metallurgical joints under the applied soldering conditions.

## Figures and Tables

**Table 1:** Nominal charge composition of the investigated solder.

Sample	Charge [wt.%]			
	Zn	Al	Ti	SiC
Zn5Al3Ti	92	5	3	-
Zn5Al3Ti + SiC nanoparticles, 3 wt.%	89	5	3	3
Zn5Al3Ti + SiC nanoparticles, 6 wt.%	86	5	3	6

**Table 2:** Parameters of the ultrasonic soldering step [4].

Ultrasonic power	[W]	400
Working frequency	[kHz]	40
Amplitude	[μm]	2
Soldering temperature	[°C]	420
Ultrasonic activation time	[s]	10

**Table 3:** Individual tensile test results retained after outlier screening of the investigated solder alloys.

Solder Alloy	Rm [MPa]	Fm [N]	A [%]
Zn5Al3Ti—1	75.2	3159.9	0.09
Zn5Al3Ti—2	45.3	1902.6	0.39
Zn5Al3Ti + 3 wt.% SiC—1	50.9	2138.4	1.11
Zn5Al3Ti + 3 wt.% SiC—2	49.9	2094.0	0.16
Zn5Al3Ti + 6 wt.% SiC—1	99.2	4168.2	0.50
Zn5Al3Ti + 6 wt.% SiC—2	85.9	3608.4	0.32

**Table 4:** Chemical composition of possible phases assigned by point EDX analysis in the Zn5Al3Ti alloy (Figure 6a).

	Al [at. %]	Si [at. %]	Ti [at. %]	Zn [at. %]	Solder Component
Spectrum 1	39.3	-	-	60.7	Eutectoid mixture (Al) + (Zn)
Spectrum 2	1.2	-	-	98.8	(Zn) solid solution
Spectrum 3	-	-	30.7	69.3	Zn_3_Ti intermetallic phase
Spectrum 4	26.9	-	22.7	50.4	Ternary intermetallic T-phase
Spectrum 5	0.6	46.3	48.8	4.3	SiTi-type phase
Spectrum 6	17.2	-	22.5	60.3	T-phase
Spectrum 7	1.8	-	25.4	69.8	Zn_3_Ti phase
Spectrum 8	15.3	-	-	84.7	Two-phase eutectoid region (Al) + (Zn)

**Table 5:** Chemical composition of possible phases assigned by point EDX analysis in the Zn5Al3Ti + 3 wt.% SiC alloy (Figure 6b).

	Al [at. %]	Si [at. %]	Ti [at. %]	Zn [at. %]	Solder Component
Spectrum 1	46.2	-	10.9	43.0	Eutectoid mixture (Al) + (Zn)
Spectrum 2	50.8	15.4	26.7	7.1	Presence of Al, Ti, Zn and additionally Si originating from SiC carbide
Spectrum 3	3.9	-	-	96.1	(Zn) solid solution with partially dissolved aluminium
Spectrum 4	15.0	-	24.3	60.8	(Zn,Al)_3_Ti phase
Spectrum 5	21.7	-	23.5	54.9	T-phase
Spectrum 6	23.2	-	-	76.8	Two-phase eutectoid regions (Al) + (Zn)
Spectrum 7	-	-	25.8	74.2	Zn_3_Ti intermetallic phase
Spectrum 8	28.2	-	24.5	47.3	T-phase

**Table 6:** Chemical composition of possible phases assigned by point EDX analysis in the Zn5Al3Ti + 6 wt.% SiC alloy (Figure 6c).

	Al [at. %]	Si [at. %]	Ti [at. %]	Zn [at. %]	Solder Component
Spectrum 1	3.1	-	-	96.9	(Zn) solid solution
Spectrum 2	18.9	-	-	81.1	Eutectoid mixture (Al) + (Zn)
Spectrum 3	19.1	-	22.6	58.3	(Zn,Al)_3_Ti phase
Spectrum 4	5.0	-	22.5	72.5	Zn_3_Ti phase
Spectrum 5	23.9	-	23.8	52.3	(Zn,Al)_3_Ti phase
Spectrum 6	-	-	27.5	72.5	Zn_3_Ti intermetallic phase
Spectrum 7	11.6	-	24.7	63.7	Zn_3_Ti + T-phase
Spectrum 8	-	47.8	49.7	2.6	SiTi phase

**Table 7:** Summary of EDS analysis of the Cu/Zn5Al3Ti + 6 wt.% SiC interfacial region.

	O [at. %]	Al [at. %]	Si [at. %]	Ti [at. %]	Cu [at. %]	Zn [at. %]	Possible Phase
Spectrum 1	4.21	0.56	6.85	-	85.99	2.40	Cu substrate
Spectrum 2	1.86	10.32	-	-	36.36	51.47	Cu–Zn intermetallic region, possibly Cu_5_Zn_8_ or CuZn
Spectrum 3	2.80	6.41	0.44	-	23.48	66.88	Zn-rich interfacial region, possibly ε(CuZn_4_) + (Zn)
Spectrum 4	4.29	27.49	0.69	22.34	0.38	44.81	Al–Ti–Zn reaction region, possibly (Al,Ti)Zn_2_
Spectrum 5	13.27	15.27	1.84	6.79	3.18	59.66	Zn5Al3Ti solder region containing Si-containing particles

**Table 8:** Summary of EDS analysis of the Zn5Al3Ti + 6 wt.% SiC/AlN interfacial region.

	Al [at. %]	Si [at. %]	Ti [at. %]	Cu [at. %]	Zn [at. %]	N [at. %]	O [at. %]	Possible Phase
Spectrum 1	6.20	0.71	0.76	4.07	84.44	-	3.81	Zn-rich solder matrix
Spectrum 2	12.21	0.27	19.45	0.42	66.14	-	1.50	Al–Ti–Zn interfacial reaction region
Spectrum 3	3.05	0.36	-	3.53	90.72	-	2.34	Zn-based solder solid solution
Spectrum 4	65.17	-	-	0.20	0.33	29.65	4.65	AlN substrate
Spectrum 5	35.79	0.28	-	3.75	52.34	-	7.83	Al–Zn-based interfacial reaction region near AlN

## Data Availability

The original contributions presented in this study are included in the article. Further inquiries can be directed to the corresponding author.
